# Challenges and perspectives of Chagas disease: a review

**DOI:** 10.1186/1678-9199-19-34

**Published:** 2013-12-19

**Authors:** Paulo Câmara Marques Pereira, Elaine Cristina Navarro

**Affiliations:** 1 Department of Tropical Diseases, Botucatu Medical School, São Paulo State University (UNESP – Univ Estadual Paulista), Av. Prof. Montenegro, Distrito de Rubião Junior, Botucatu, São Paulo State, Brazil; 2 Eduvale School of Avaré, Avaré, São Paulo State, Brazil

**Keywords:** Control of Chagas disease, Epidemiology, Transmission

## Abstract

Chagas disease (CD), also known as American trypanosomiasis, is caused by the flagellated protozoan *Trypanosoma cruzi*, and affects an estimated 8 to 10 million people worldwide. In Latin America, 25 million people live in risk areas, while in 2008 alone, 10,000 CD-related deaths were reported. This review aimed to evaluate the challenges of CD control, future perspectives, and actions performed worldwide to control expansion of the disease and its impact on public health in Latin America.

## Introduction

Chagas disease (CD), also known as American trypanosomiasis, is caused by the flagellated protozoan *Trypanosoma cruzi*, and affects an estimated 8 to 10 million people worldwide [1]. In Latin America, 25 million people live in risk areas whereas, in 2008 alone, 10,000 CD-related deaths were reported. The incidence of the disease is high in rural areas where environmental conditions favor the installation and breeding of triatomine bugs [2].

In the 1970s, 100,000 new cases of CD were recorded per year in Brazil. This annual rate decreased to 10,000 after the implementation of effective campaigns for the control of vector transmission (the main route of acquisition of the disease). Today, about 3 million people are estimated to be infected, but this number could be much higher since most individuals do not exhibit symptoms and are classified as carriers of the indeterminate form of CD [3].

This review aimed to evaluate the challenges of CD control, future perspectives, and actions performed worldwide to mitigate expansion of the disease and its impact on public health in Latin America.

## Review

### Mechanisms of transmission

The two main transmission routes of CD include common and uncommon or accidental transmission. The most common routes are vector transmission (bite of triatomine bugs), transfusion (transfusion of blood contaminated with *T. cruzi*), oral transmission (ingestion of foods or beverages contaminated with triatomine feces), and vertical or congenital transmission (parasite crossing the placental barrier). Uncommon or accidental modes of transmission include transmission during organ transplantation, ingestion of maternal milk contaminated with the protozoan, laboratory accidents, contamination of foods with secretions from the anal glands of marsupials harboring the parasite, bites of contaminated arthropods (demonstrated experimentally), and sexual relations (contamination of men who have had sexual contact with infected women during their menstrual period) [4].

Vector transmission is the classical form of CD acquisition. This transmission route has the largest impact in Latin American countries and is also responsible for the maintenance of the disease. In this case, the insect vector is contaminated by feeding on the blood of an infected host and defecates at the site of the bite wound. Parasites then enter the bloodstream and invade cells of the monophagocytic system. Another possibility of transmission is contamination of the proboscis of the vector with its own feces or with feces from another contaminated vector [4,5] (Additional file 1http://www.youtube.com/watch?v=1ais69H0li8).

The following triatomine species can transmit CD: *Triatoma sordida*, *Triatoma pseudomaculata*, *Triatoma tibiamaculata*, *Triatoma arthurneivai*, *Triatoma brasiliensis*, *Triatoma dimidiata*, *Panstrongylus megistus*, *Panstrongylus geniculatus*, *Panstrongylus diasi*, *Rhodnius neglectus*, *Rhodnius prolixus*, *Rhodnius megistus*, and *Rhodnius domesticus*. However, the main species related to CD transmission in Brazil is *Triatoma infestans*[6].

In the 1950s, the Brazilian government implemented vector control campaigns in some regions of the country; however, only in the 1980s were these campaigns extended to the whole territory. In 1991, the National Health Foundation (FUNASA) assumed the control of all endemic diseases. At the same time, the South American countries where two-thirds of CD carriers in the Americas are concentrated (Argentina, Brazil, Chile, Uruguay, Paraguay, Bolivia and Peru) started an international cooperation program, the South Cone Initiative, whose objective was to control the vector and transfusion transmission [7-9]. The success of the campaigns for vector eradication was so impressive that some countries have been certified free of vector transmission, including Uruguay (1997), Chile (1999), and Brazil (2006). The Pan American Health Organization (PAHO) certificate declaring an area free of transmission of the *Triatoma infestans* vector does not signify complete interruption of transmission, but rather effective control [10-12]. Furthermore, improvement in the socioeconomic conditions of the Brazilian population in recent decades has provided dwellings that were less favorable to transmission of the vector, thus contributing to the control of this disease.

Another goal achieved by the South Cone Initiative was the mandatory serological screening of blood banks, including 100% of public blood banks and 80% of private blood banks in Argentina, and all blood banks in Brazil, Chile and Uruguay. On the other hand, Paraguay, Bolivia and Peru continue to fight against the disease, but have not yet reached the targets of the program [8].

A number of studies involving blood donors from the whole country have been conducted in an attempt to determine the seroprevalence of chagasic infection in Brazil. Sobreira *et al.*[13] studied 3,232 blood donors from the Iguatu Blood Center (Ceará state) and found that 61 (1.9%) presented serology positive for CD. At the São Lucas Hospital of the Pontifical Catholic University of Rio Grande do Sul, 8,228 samples were tested by different methods (ELISA, hemagglutination and indirect immunofluorescence) and revealed a seroprevalence ranging from 0.4 to 0.5% [14]. In another study conducted in Rio Grande do Sul state, Fitarelli and Horn [10] observed that the prevalence of CD in the southern region was lower than the national average (0.41% versus 0.61%). Silva and Silva [15] found a CD seroprevalence of 1.2% at the Hemominas Foundation (Patos de Minas, Minas Gerais state).

A major problem encountered in seroprevalence studies was the high incidence of inconclusive serological reactions. At the Blood Center of Pernambuco (Hemope), 743,529 blood donations made between 2002 and 2007 were evaluated; of these, 1,264 were negative for CD, including 39.7% reactive samples and 60.3% inconclusive samples [16]. At the Uberaba Blood Center, 52% of all non-negative serological reactions were inconclusive [17]. Navarro *et al.*[18], while analyzing non-negative serological reactions at the Botucatu Blood Center, found 0.5% non-negative and 35.4% inconclusive.

Picka *et al.*[19] investigated inconclusive serological reactions by different methods (ELISA, indirect hemagglutination, indirect immunofluorescence and TESA-cruzi immunoblotting) and concluded that TESA-cruzi is the best method to confirm positive serology in individuals with more than two inconclusive reactions.

The success of vector transmission control can be observed in the studies cited above and corroborates data reported by Dias [20] and Moraes-Souza *et al.*[21] showing a reduction in the seroprevalence of CD among blood donors from 7 to 0.6% between 1970 and 2006.

Oral transmission has gained much attention in the past decade. Until 2004, few CD-related studies on contaminated foods and beverages have been published. However, after an initial outbreak that occurred in the state of Santa Catarina in 2005, new outbreaks have been reported, especially in the northern region where the consumption of fresh açaí berries is frequent. In the state of Pará alone, oral transmission was responsible for 178 cases of the acute form of CD in 2006. As a consequence, the Brazilian authorities implemented guidelines of good manufacturing practices and mandatory pasteurization of beverages and foods related to the oral transmission of CD [22].

In countries with currently successful vector control, congenital or vertical transmission has become the focus of attention. Although the congenital transmission rate is low (4-8%), it may be responsible for maintaining the transmission cycle among young individuals, particularly among women of childbearing age [23].

Over the last two decades, congenital transmission has received special attention in different countries that are not endemic for CD, since various cases were diagnosed in Latin American women. In light of the migration of Latin American women to European countries and to North America, some countries – including Spain and USA – have established serological screening of pregnant women for CD in some cases. In Brazil, only a few studies on CD in pregnant women are available. Furthermore, serological screening for CD is not part of routine prenatal care, even in historically endemic areas.

In a study conducted at seven hospitals in Madrid, Spain, involving 3,839 pregnant Latin American women, the seroprevalence of CD was 3.96% and the rate of congenital transmission was 2.6%, demonstrating the impact of this route of transmission [24].

Romero *et al.*[25] studied 1,730 women of childbearing age from the rural population of Carapari, Bolivia, and found that 64.5% were seroreactive for CD. In addition, an examination of 468 newborns of seroreactive women found a congenital transmission rate of 4% (12/299). To confirm the diagnosis of congenital transmission, the infants were monitored up to 12 months of age, since maternal IgG antibodies transferred through the placenta can be present until 7 months of age.

Araujo *et al.*[26] conducted an epidemiological study of CD involving pregnant women from the Brazilian town of Pelotas (Rio Grande do Sul state). A total of 351 umbilical cord serum samples obtained from the parturients were analyzed and only one case was positive. For screening purposes, samples were collected from all household members and animals living in the residence of the infected mother and only the mother was seroreactive. The women reported having received a blood transfusion during childhood, which probably was the route of transmission.

According to some studies, congenital transmission is due to large quantities of circulating parasites in maternal blood before delivery. Reduced interferon gamma (IFN-γ) production during pregnancy is related to higher rates of contaminated neonates in contrast to higher monocyte activation in the mothers who did not transmit the parasite to their children [27,28]. As a consequence, congenital transmission is directly related to maternal parasite burden.

According to Fretes and Kemmerling [29], during pregnancy the maternal immune system potentiates cellular and molecular recognition, cell-to-cell communication, and cell repair in order to protect the fetus whose immune system is still developing [28]. Nevertheless, the maternal immune system is not always efficient. Duaso *et al.*[30] showed that apoptosis is a normal event during pregnancy as part of placental tissue renewal, eliminating undesired cells such as inflammatory cells. However, in women infected with *T. cruzi*, apoptosis may affect healthy cells and favor the passage of the parasite through chorionic villi.

Brutus *et al.*[31] studied 513 mothers and 516 infants from Bermejo (southern Bolivia) where vector transmission is absent and observed a CD prevalence of 33.9% among mothers, particularly those aged 26 to 35 years. In that study, the congenital transmission rate was 5.2%, but the severity of the disease was low in these infants.

Since 1987, the World Health Organization (WHO) has recommended preventive chemotherapy (benznidazole or nifurtimox) for pregnant women with CD despite its high toxicity, to reduce parasite burden as well as the risk of congenital transmission [27].

According to Bern and Montgomery [32], the control of congenital transmission is the main measure for preventing CD in countries where insect vectors are absent.

A study involving all pregnant women seen at three maternity wards in Santa Cruz de la Sierra (western Bolivia) was conducted to determine congenital transmission rates. The Stat-Pak Assay®, a rapid test for the diagnosis of CD, and a third-generation ELISA (Chagatest®) were used. A total of 257 newborns, including 111 infected infants of seroreactive mothers, 68 negative infants born to positive mothers and 78 negative neonates of negative mothers, were studied. All newborns with confirmed positive serology were treated with benznidazole and presented negative serology after 6 months, demonstrating the efficacy of treatment during the acute phase [33].

Some Latin American countries such as Paraguay, Uruguay and some regions in Argentina have already implemented prenatal screening for CD. In Brazil, screening of pregnant women is not part of the national program; however, the Association of Parents and Friends of Exceptional Children (APAE) performs screening tests for CD in the states of Mato Grosso do Sul and Goiás [34].

According to De Rissio *et al.*[35] control measures of congenital transmission should be established as a public health priority in all endemic regions and for pregnant women originating from these areas, since evidence indicates congenital transmission to be the current main mode of transmission that may lead to global dissemination of the disease.

The diagnosis of congenital transmission is important for public health since, although diagnosed pregnant women tend to be in the chronic symptomatic or asymptomatic phase for which no proven effective treatment exists, their children are in the acute phase for which treatment and a definitive cure are available. According to González-Tomé *et al.*[23] an estimated 2 million women of childbearing age are infected with the protozoan *T. cruzi* in the Americas while the rate of congenital transmission ranges from 4 to 8%; thus, the number of infected children may reach 15 million.

### Determinants of infection

One of the main determinants of *T. cruzi* infection is the initial inoculum. The different triatomine species that can transmit CD show distinct patterns of parasite elimination in feces. Borges-Pereira *et al.*[36] evaluated eight triatomine species infected with *T. cruzi* during feeding and observed an average of 40 parasites per evacuation. When the mean number of parasites eliminated per species was analyzed, *P. megistus* was found to be the most effective (232) and *T. pseudomaculata* the least effective (51 parasites).

Some decades ago, reinfection was another problem encountered in historically endemic areas such as the interior of the states of Minas Gerais, Pernambuco and São Paulo, which was mainly due to precarious housing conditions that favored the infestation of dwellings and attracted triatomine bugs to peridomestic areas. Today, this is an important characteristic of the Brazilian Amazon region where biological (wide diversity of wild reservoirs and vectors) and social determinants (intense internal migration) favor the introduction of multiple parasite strains. Over the last 300 years, human invasion of wild environments, deforestation and cattle farming have favored the establishment of domestic and peridomestic cycles [37]. There are few reports of patients with chronic CD in these regions, but more than 100 cases of the acute form of the disease have been registered, particularly cases of oral transmission [38,39].

Until the 1980s, *T. cruzi* strains were subdivided into three groups (zymodemes): I, II and Z3, with groups I and Z3 (III) including wild strains and group II including domestic strains. With the advances in molecular biology techniques, it can now be confirmed that the parasite presents a great genetic diversity and can be classified into six discrete typing units, which possess common immunological and molecular markers. Strains I (wild) and II (domestic) are pure and strains III to VI are hybrids [40,41].

The initial immune response of the host seems to be crucial for the clinical progression of CD. Some authors defend the proposition that the initial increase in IFN-γ, followed by an increase in IL-10 (both cytokines produced by CD4+ lymphocytes), is responsible for the clinical course of the indeterminate form of the disease [42,43].

### Clinical phases

The incubation period of *T. cruzi* ranges from 3 to 112 days depending on the mode of infection [44]. The acute phase lasts 4–8 weeks and is generally asymptomatic or oligosymptomatic. In the latter case, the disease manifests as a self-limited febrile illness whose symptoms may occur 1 to 2 weeks after vector contamination or within a few months after transfusion of contaminated blood. In symptomatic cases, prolonged fever, malaise, hepato- and/or splenomegaly, lymphadenomegaly, localized or generalized subcutaneous edema, and signs at the site of parasite entry (Romaña’s sign or chagoma inoculation) are observed. Cardiac and neurological alterations are rare. The mortality rate ranges from 5 to 10% in acute cases, generally involving children who die of myocarditis and/or myeloencephalitis [40,45].

Most acute cases of CD in Brazil are registered in the northern states, but there are still sporadic reports of acute cases in the southeastern region of the country. In 2006, a 6-year-old child living in the rural area of Itaporanga died after 27 days of nonspecific symptoms and erroneous diagnoses. This case illustrates why CD is considered the most neglected of the neglected tropical diseases since, although this child lived in a historically endemic area for CD, at no time was the diagnosis of the acute phase taken into consideration [46].

In a study conducted at the University Hospital of Botucatu, Geraix *et al.*[47] observed that more than 70% of patients with CD had the indeterminate form, followed by the digestive, cardiac and mixed form [46]. The course of the chronic form of the disease is variable, with about 60% of parasitized individuals presenting the indeterminate form (no symptoms) and 20 to 40% developing the cardiac and/or digestive form [48].

The indeterminate form of CD shows the best clinical prognosis for chronic patients, since there is serological demonstration of the presence of the parasite but no cardiac or digestive involvement, a course that can continue until the end of life. The main problem encountered by physicians and patients in these cases is the uncertainty. It is very common that the patient believes his heart will “swell” at any time and that he will lose the capacity to perform daily activities. Since there is no safe marker of disease progression, this difficult situation is likely to continue.

According to Maya *et al.*[49] there are four main mechanisms underlying the pathogenesis of the cardiac form of CD: parasite-induced myocardial damage, immune-mediated myocardial damage, cardiac dysautonomia, and cardiovascular anomalies and ischemia.

In an experimental study, Coura [50] demonstrated a reduction in cardiopathy when animals were treated with anti-trypanosomatid drugs in order to reduce parasite burden. The author showed that parasite persistence in host tissues plays an important role in myocardial aggression.

According to Marin-Neto *et al.*[51] the cardiac form of CD is characterized by inflammatory infiltration, cell death and reparative interstitial fibrosis, events that lead to disturbances in the cardiac conduction system (intraventricular and atrioventricular blockade, sinus node dysfunction, and ventricular arrhythmia) and myocarditis, causing electrical instability (atrial arrhythmia), reduced contractility (intracavitary thrombosis and heart failure) and microvascular disturbances (tip aneurism) which can result in sudden death. Alterations in the intracardiac nervous system can cause atypical chest pain and also sudden death.

Digestive problems are observed in one-third of chagasic patients and usually result in dilatation of the gastrointestinal tract. According to Lescure *et al.*[52] the digestive form is responsible for the development of mega-syndromes of the esophagus (dysphagia, chest pain, and regurgitation) and colon (chronic constipation, abdominal pain, and obstruction). Involvement of the esophagus includes the loss of esophageal peristalsis and lack of relaxation of the lower sphincter during swallowing, impairing deglutition and causing progressive dilatation of the organ. Esophageal dysfunction may also be associated with alterations in intestinal transit. This form of the disease is characterized by the destruction of ganglionic neurons and increasingly slower intestinal transit, leading to muscle hypertrophy and, in more exacerbated cases, to dilatation of the organ [53].

According to Oliveira *et al.*[54], there are an estimated 300,000 individuals with megacolon, the main symptoms of which are constipation and fecal impaction. Silva *et al.*[55], reported alterations in intestinal secretion, absorption and motility in these patients, which culminate in mega-syndromes (dilated area upstream from the achalasic segment). Patients with megacolon can also present chronic constipation, abdominal pain, volvulus, obstructions, and intestinal perforations.

### Diagnosis

The clinical diagnosis of CD can only be confirmed during the acute phase if there are signs of parasite entry such as Romaña’s sign and/or inoculation chagoma. This phase is characterized by high parasitemia, a fact that permits a parasitological diagnosis. The thick drop test has been frequently used in epidemiological field studies due to its low cost and easy visualization of the parasite; however, rapid serological tests such as the Stat-Pack assay have attracted the attention of researchers since they permit a diagnosis of chronic carriers of the disease [33].

The chronic phase of CD is characterized by low parasitemia, which hampers the efficiency of direct identification of the parasite. Therefore, other methods – namely parasitological (xenodiagnosis), serological (ELISA, indirect hemagglutination, indirect immunofluorescence, chemiluminescence, and TESA-blot), and molecular biology (PCR) – are used during this phase.

The xenodiagnosis technique was introduced by Brumpt in 1914 (natural xenodiagnosis) and consists of the direct application of triatomine nymphs on the skin surface, which caused discomfort and refusal of the patients to undergo this test. In the 1940s, Romana and Gil introduced artificial xenodiagnosis, which avoids direct contact of the individual with the bug [19].

ELISA has been used for many years as the gold standard for diagnosing CD. In this method, *T. cruzi*-specific antigens are fixed on an ELISA plate (sensitization phase). Next, the patient serum is incubated with a primary antibody, followed by incubation with a conjugated antibody for the formation of an immunocomplex. Finally, a chromogen solution is added to the reaction for color production, which can be analyzed in an ELISA reader at a given wavelength [56].

The chemiluminescence test is an enzyme immunoassay for the qualitative detection of anti-*T. cruzi* IgG antibodies. The test requires two steps: samples and diluents are mixed with paramagnetic microparticles coated with recombinant *T. cruzi* antigens (FP3, FP6, FP10, and TcF); then, human IgG antibodies are labeled with acridine and the chemiluminescence reaction is measured in relative light units.

Indirect hemagglutination was introduced by Cerisola *et al*. [57]. This method uses stabilized bird erythrocytes that are sensitized by binding to highly purified *T. cruzi* antigens. Agglutination is observed when these antigens react with antibodies present in the patient’s serum. The reaction is defined as positive when the red blood cells settle in the bottom of the microplate well, forming a uniform network that is sometimes partially retracted at the borders [19].

The TESA-blot is a Western blot method that uses TESA (antigens excreted from trypomastigotes) and *T. cruzi* strain Y (TESA-blot). Positive reactions are indicated by the presence of bands at a molecular weight of 120–200 kDa [19].

Blood culture uses liver-infusion tryptose (LIT) medium for the incubation of a patient’s blood. The culture is analyzed for up to 180 days and the observation of flagellate forms of *T. cruzi* is defined as a positive result [58].

### Treatment

Chagas disease is considered the most neglected of the neglected tropical diseases, a fact that can be demonstrated within the context of treatment. In the 1970s, two drugs that act by forming free radicals and/or electrophilic metabolites were made available on the market. In Brazil, only benznidazole is currently used and its efficacy is observed during the acute phase of CD [59]. The lack of effective drugs for treatment of the chronic phase, particularly the chronic indeterminate form, has brought anguish for patients and their doctors who have taken care of them for decades. Benznidazole acts through three effector mechanisms: trypanocidal action (formation of covalent bonds with *T. cruzi* macromolecules such as DNA and cytochrome P450); increase in phagocytosis and parasite lysis through an IFN-γ-dependent mechanism; and, finally, inhibition of parasite growth by blocking NADH-fumaratereductase. Despite its efficacy in the treatment of the acute phase, benznidazole has several side effects ranging from hypersensitivity reactions to bone marrow depression and peripheral polyneuropathy [60].

Among the new drugs being tested, some are already in clinical trials. One of these drugs, posaconazole, has shown promising results in the treatment of patients in the acute and chronic phase. This drug was found to be effective in eliminating amastigotes from cardiac cells. Some studies have used combinations of drugs such as benznidazole, nifurtimox, benznidazole and allopurinol, among others [61].

The research group of the School of Medicine of Ribeirão Preto (FMRP/USP) is developing new drug candidates based on the capacity of nitric oxide to kill the parasite [62]. Guedes *et al.*[63] tested treatment with *trans*-[RuCl([15] aneN4)NO]2+ *in vitro* and *in vivo* and demonstrated that this drug induces the release of nitric oxide inside macrophages, exerting a significant trypanocidal effect [63]. When administered in combination with benznidazole, this substance prevents death by reducing parasitemia and cardiac inflammation. Another study conducted by the same group used *cis*-[Ru(NO)(bpy)2 L]X*n* and showed that the drug exerts a more effective trypanocidal activity at a lower dose than benznidazole [64].

Despite progress in the identification of new drug candidates against CD, the therapeutic proposal has remained the same for more than 40 years and there is no effective treatment for the chronic phase of the disease.

## Conclusions

Several Latin American countries have joined efforts to control the different routes of CD transmission, but an effective control in the Americas is still a distant dream. In Brazil, the control of vector and transfusion transmission has been shown to be effective, but serological screening of pregnant women from historically endemic areas for CD is not yet mandatory.

Another important step related to CD is the development of new drugs. The two currently available drugs (benznidazole and nifurtimox) are effective in treating the acute phase of the disease, but not the chronic phase. In addition, the side effects of these drugs are severe and are responsible for the fact that many patients abandon treatment. One of the main achievements in CD treatment was the development of dispersible tablets that are used for treating children, since in the past the parents had to fractionate the medication into as many as 12 parts, favoring treatment failure due to incorrect doses. The pediatric medication is produced exclusively by the Pharmaceutical Laboratory of the state of Pernambuco (LAFEPE, Brazil) supported by the Drugs for Neglected Disease Initiative (DNDi).

Another important national achievement is being made by researchers at FMRP, where a new drug consisting of a combination of benznidazole and nitric oxide is being patented. In addition to reducing the parasite burden in mice, this drug has been shown to increase survival by 100%.

Immunological studies are being conducted at various universities around the world in an attempt to understand the immune response of patients with indeterminate CD, since parasitemia is maintained under control in these patients. A precise understanding of these mechanisms may contribute to the development of new drugs. At the Botucatu Medical School (FMB/UNESP), various research groups from different fields are studying CD. The authors of the present study are part of the research group at the Laboratory of Tropical Diseases of FMB, where immunological, molecular and metabolic aspects of the disease are investigated. At present, one of the groups is analyzing the relationship of the lipid profile and nutritional status of patients with CD with proinflammatory cytokines and nitric oxide. Preliminary results are available and will be published soon.

Although CD is a centuries-old disease and many efforts have been made in Brazil to control the main routes of transmission, many aspects remain unclear, a situation that requires further studies, particularly those focusing on immunological aspects and on investigating new drugs. Albeit successful measures have been established in Brazil, there are still cases of young individuals with CD. Hence, the price of maintaining these achievements will be eternal vigilance.

## Competing interests

The authors declare that there are no competing interests.

## Supplementary Material

Additional file 1 **Video showing the life cycle of *T. cruzi *** **in the human host.** Available at: http://www.youtube.com/watch?v=1ais69H0li8. Source: videosINBEB; Dirceu Esdras Teixeira, Marlene Benchimol, Paulo Crepaldi and Wanderley de Souza. “Animated life cycle of *T. cruzi* in the human host.” YouTube. Published: August 30, 2012. Accessed: November 7, 2013. Click here for file
